# High throughput circRNA sequencing analysis reveals novel insights into the mechanism of nitidine chloride against hepatocellular carcinoma

**DOI:** 10.1038/s41419-019-1890-9

**Published:** 2019-09-10

**Authors:** Dan-dan Xiong, Zhen-bo Feng, Ze-feng Lai, Yue Qin, Li-min Liu, Hao-xuan Fu, Rong-quan He, Hua-yu Wu, Yi-wu Dang, Gang Chen, Dian-zhong Luo

**Affiliations:** 1 Department of Pathology, First Affiliated Hospital of Guangxi Medical University, Nanning, China; 2 Pharmaceutical College, Guangxi Medical University, Nanning, China; 3 Department of Toxicology, Pharmaceutical College, Guangxi Medical University, Nanning, China; 4 Department of Medical Oncology, First Affiliated Hospital of Guangxi Medical University, Nanning, China; 5 Department of Cell Biology & Genetics, School of Preclinical Medicine, Guangxi Medical University, Nanning, China

**Keywords:** Cancer therapy, Non-coding RNAs

## Abstract

Nitidine chloride (NC) has been demonstrated to have an anticancer effect in hepatocellular carcinoma (HCC). However, the mechanism of action of NC against HCC remains largely unclear. In this study, three pairs of NC-treated and NC-untreated HCC xenograft tumour tissues were collected for circRNA sequencing analysis. In total, 297 circRNAs were differently expressed between the two groups, with 188 upregulated and 109 downregulated, among which hsa_circ_0088364 and hsa_circ_0090049 were validated by real-time quantitative polymerase chain reaction. The in vitro experiments showed that the two circRNAs inhibited the malignant biological behaviour of HCC, suggesting that they may play important roles in the development of HCC. To elucidate whether the two circRNAs function as “miRNA sponges” in HCC, we identified circRNA-miRNA and miRNA-mRNA interactions by using the CircInteractome and miRwalk, respectively. Subsequently, 857 miRNA-associated differently expressed genes in HCC were selected for weighted gene co-expression network analysis. Module Eigengene turquoise with 423 genes was found to be significantly related to the survival time, pathology grade and TNM stage of HCC patients. Gene functional enrichment analysis showed that the 423 genes mainly functioned in DNA replication- and cell cycle-related biological processes and signalling cascades. Eighteen hubgenes (SMARCD1, CBX1, HCFC1, RBM12B, RCC2, NUP205, ECT2, PRIM2, RBM28, COPS7B, PRRC2A, GPR107, ANKRD52, TUBA1B, ATXN7L3, FUS, MCM8 and RACGAP1) associated with clinical outcomes of HCC patients were then identified. These findings showed that the crosstalk between hsa_circ_0088364 and hsa_circ_0090049 and their competing mRNAs may play important roles in HCC, providing interesting clues into the potential of circRNAs as therapeutic targets of NC in HCC.

## Introduction

Liver cancer is the fourth leading cause of cancer-associated deaths globally, of which the most common histological type is hepatocellular carcinoma (HCC)^1^. Although surgery is the main treatment for HCC, most patients have developed advanced HCC at the time of diagnosis due to the atypical symptoms in the early stages, missing the ideal operation time. For patients with unresectable advanced HCC, pharmacotherapy is the primary therapeutic strategy^2^. Nevertheless, due to drug resistance^3^ and high rates of adverse side effects^4^, most conventional chemotherapeutic agents fail to produce satisfactory outcomes for HCC patients. Thus, the development of treatment methods that produce milder adverse side effects and can effectively prolong the survival time of patients with HCC has been the focus of research.

Natural products have received increasing interest in exploration of novel antitumour therapeutic remedies because of their low side effects and extensive bioactivities. Nitidine chloride (NC), a major active ingredient that was isolated from the roots of the traditional Chinese medicine herb *Zanthoxylum nitidum* (Roxb) DC, has been demonstrated to have anticancer effects in human malignant tumours, including HCC^5–7^. However, the mechanisms of action of NC against HCC remain largely unclear to date. Our research group has reported that NC may exert its anti-HCC role by targeting TOP1A and TOP2A^8^, but whether NC can fight HCC by other targets is still worth exploring.

Circular RNAs (circRNAs), a class of transcripts without a 3′ tail and or 5′ cap, are naturally occurring endogenous ncRNAs with a covalently closed loop. Cumulative studies have highlighted the important roles of circRNAs in cancer occurrence and progression^9–11^. In our previous study, we identified two circRNAs (hsa_circRNA_100291 and hsa_circRNA_104515) that may be involved in the pathogenesis of HCC and revealed their prospects as therapeutic targets for anticancer drugs^12^. However, more studies are needed to verify the role of circRNAs in the pharmaceutical treatment of HCC.

In this study, we performed an RNA sequencing (RNA-seq) analysis of circRNAs based on three pairs of NC-treated and NC-untreated HCC xenograft tumour tissues to identify circRNAs that may be potential targets for NC against hepatocarcinogenesis. Following the corroboration of the differently expressed circRNAs by real-time quantitative polymerase chain reaction (RT-qPCR) and the exploration of the biological function of the circRNAs by in vitro experiments, a circRNA-miRNA-mRNA network was constructed by using a website-based computational biology strategy. Simultaneously, a weighted gene co-expression network analysis (WGCNA) along with Gene Ontology (GO) functional annotations and Kyoto Encyclopedia of Genes and Genomes (KEGG) analyses were conducted to explore the underlying mechanisms of action of the circRNAs in HCC in depth. Altogether, these analyses provide novel insights into the possibility of circRNAs serving as targets of NC anti-HCC.

## Results

### NC inhibits proliferation and induces apoptosis of HCC cells in vitro

**Fig. 1:**
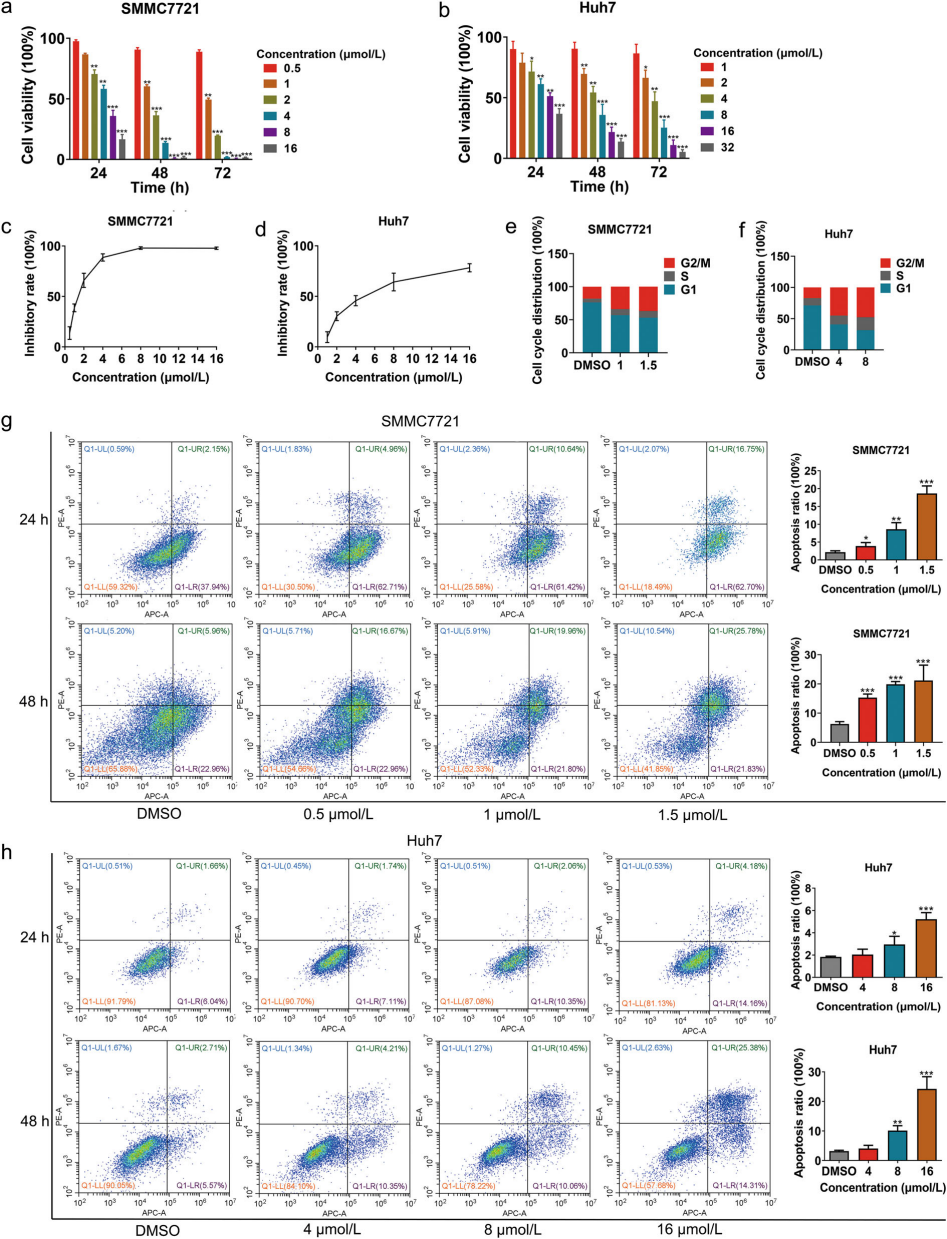
Nitidine chloride (NC) inhibits viability and induces apoptosis of hepatocellular carcinoma (HCC) cells. Cell viability of SMMC7721 (**a**) and Huh7 (**b**) following NC treatment for 24, 48 and 96 h. Inhibition rate of SMMC7721 (**c**) and Huh7 (**d**) following NC treatment for 48 h; Cell cycle distribution of SMMC7721 (**e**) and Huh7 (**f**) following NC treatment for 24 h; Cell apoptosis of SMMC7721 (**g**) and Huh7 (**h**) following NC treatment for 24 and 48 h. DMSO dimethyl sulfoxide

To elucidate the inhibitory effect of NC on HCC cell proliferation, we performed a Cell Counting Kit-8 (CCK8) assay. As shown in Fig. 1a, b, NC caused a time- and dose-dependent decrease in the growth of SMMC7721 and Huh7 cells, with the 50% inhibitory concentration (IC_50_) of 1.332 μmol/L in SMMC7721 and of 5.006 μmol/L in Huh7 after treatment of NC for 48 h, respectively (Fig. 1c, d). Cell cycle analysis showed that 24-h NC treatment induced G2/M phase arrest in SMMC7721 and Huh7 cells in a dose-dependent manner (Fig. 1e, f), indicating that NC inhibited cell growth by G2/M phase arrest.

To determine whether NC could induce apoptosis in HCC cells, we harvested SMMC7721 and Huh7 cells treated with various concentrations of NC for 24 and 48 h for flow-cytometry analysis. The results showed that NC promoted late apoptosis of HCC cells (Fig. 1g, h).

### NC suppresses migration of HCC cells in vitro

**Fig. 2:**
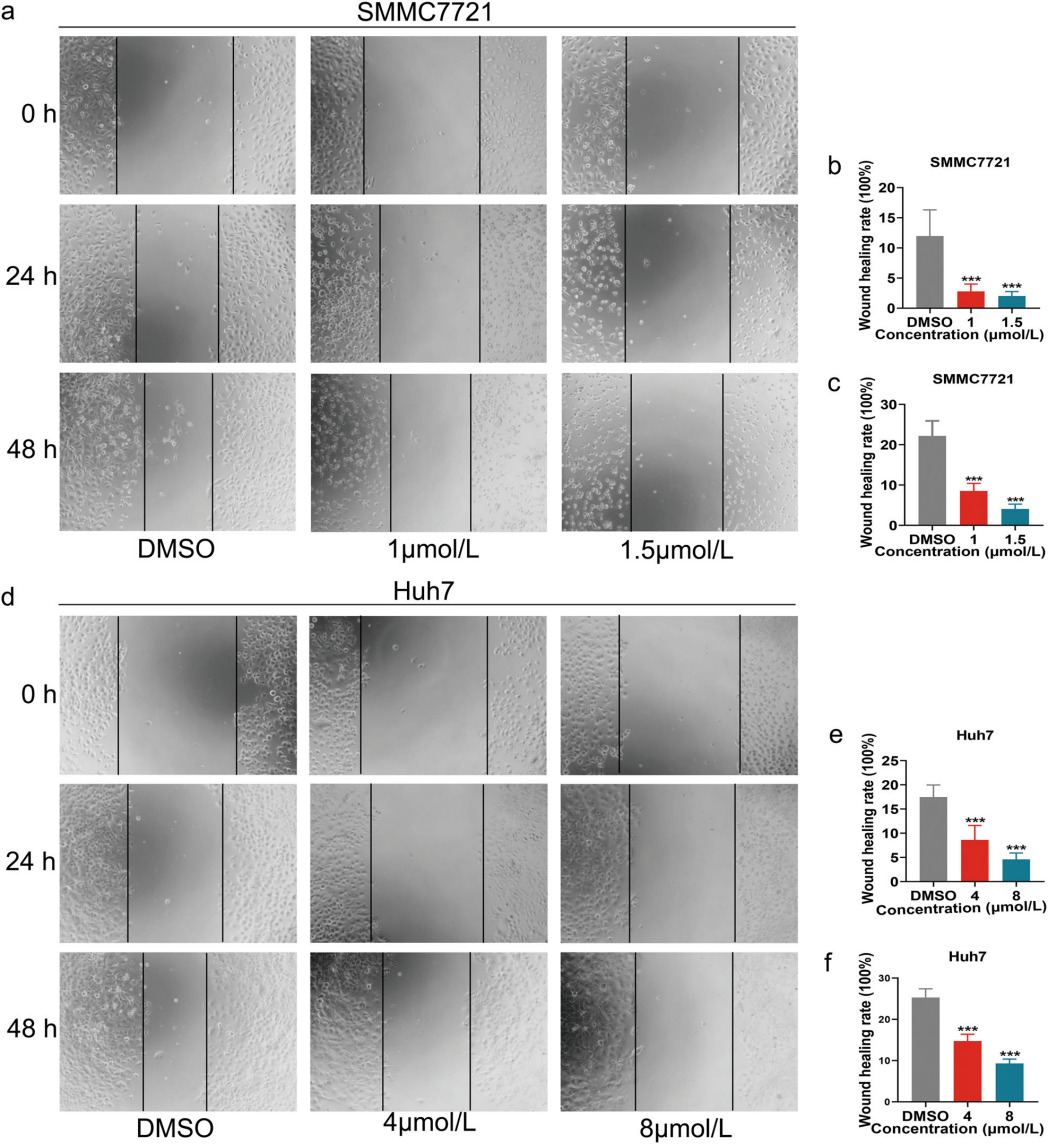
Nitidine chloride (NC) inhibits migration of HCC cells. Cell migration of SMMC7721 (**a**–**c**) and Huh7 (**d**–**f**) following NC treatment for 24 and 48 h. DMSO dimethyl sulfoxide

A wound-healing assay was conducted to determine the effect of NC on HCC cell migration. The results showed that NC treatment repressed migration of SMMC7721 and Huh7 cells in a time- and dose-dependent manner (Fig. 2).

### Differently expressed circRNAs based on RNA-seq analysis and RT-qPCR

**Fig. 3:**
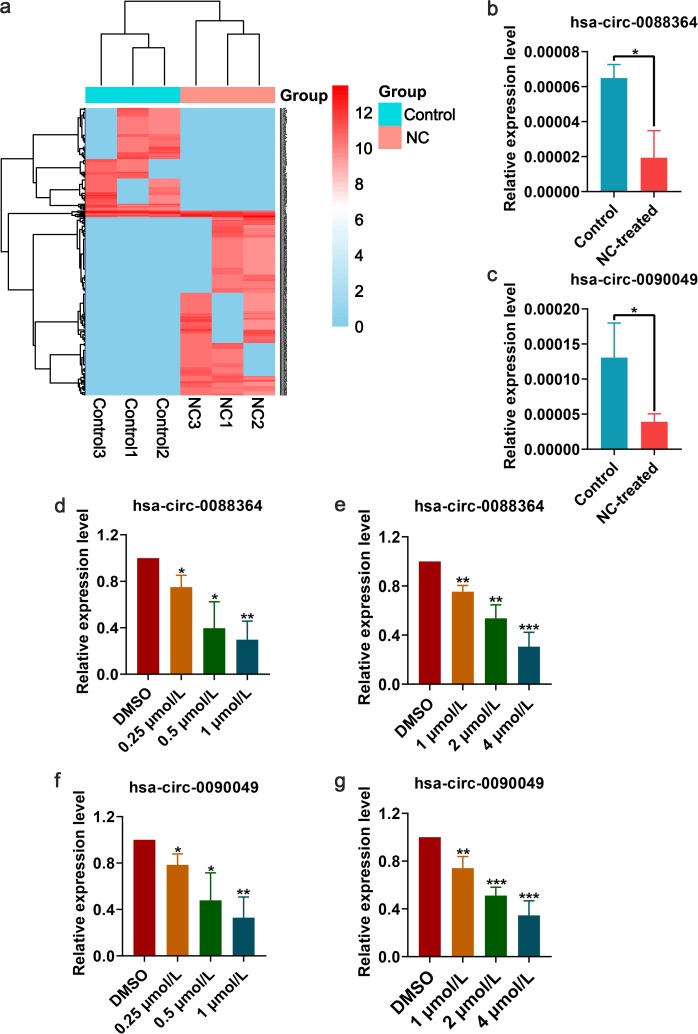
Differentially expressed circRNAs between control and nitidine chloride (NC)-treated groups. **a** Hierarchical cluster analysis of 297 circRNAs that were differently expressed between three pairs control and NC-treated xenograft hepatocellular carcinoma (HCC) tumour tissues. Corroboration of hsa_circ_0088364 (**b**) and hsa_circ_0090049 (**c**) by real-time quantitative polymerase chain reaction (RT-qPCR) in three pairs control and NC-treated HCC xenograft tumour tissues. Expression of hsa_circ_0088364 in SMMC7721 (**d**) and Huh7 (**e**) cells treated and untreated with NC. Expression of hsa_circ_0090049 in SMMC7721 (**f**) and Huh7 (**g**) cells treated and untreated with NC. Data are represented as mean +/− SD. DMSO dimethyl sulfoxide

Three pairs of NC-treated and NC-untreated HCC xenograft tumour tissues were collected for RNA-seq analysis of circRNAs. Compared with those of the NC-untreated group, the tumour volumes in the NC-treated group were significantly decreased (*p*-value < 0.05)^13^. A total of 297 circRNAs were differently expressed between the two groups, with 188 upregulated and 109 downregulated (Fig. 3a). Three circRNAs (hsa_circ_0088364, hsa_circ_0090049 and hsa_circ_0102434) were selected for RT-qPCR validation in the three pairs xenograft tumour tissues, and the expression changes of hsa_circ_0088364 and hsa_circ_0090049 were consistent with the RNA-seq results (Fig. 3b, c). Additionally, the cell lines SMMC7721 and Huh7 treated or not with NC were used and a dose-dependent inhibitory effect of NC on the expression of hsa_circ_0088364 and hsa_circ_0090049 was corroborated (Fig. 3d–g), indicating that the two circRNAs could be targets of NC. The basic characteristics of the two circRNAs are shown in Table 1.

**Table 1:** Main characteristics of the two circRNAs

circRNA	Position	Strand	Best transcript	Gene symbol	miRNA binding element
hsa_circ_0088364	chr9:125047447-125048225	+	NM_138777	MRRF	miR-1299, miR-140-3p, miR-145, miR-548l, miR-1252, miR-548c-3p, miR-766, miR-942, miR-1276, miR-663b, miR-326, miR-330-5p, miR-599, miR-1273, miR-620
hsa_circ_0090049	chrX:21869626-21871621	+	NM_015884	MBTPS2	miR-605, miR-548c-3p, miR-223, miR-665

### Biological effects of hsa_circ_0088364 and hsa_circ_0090049 in HCC

**Fig. 4:**
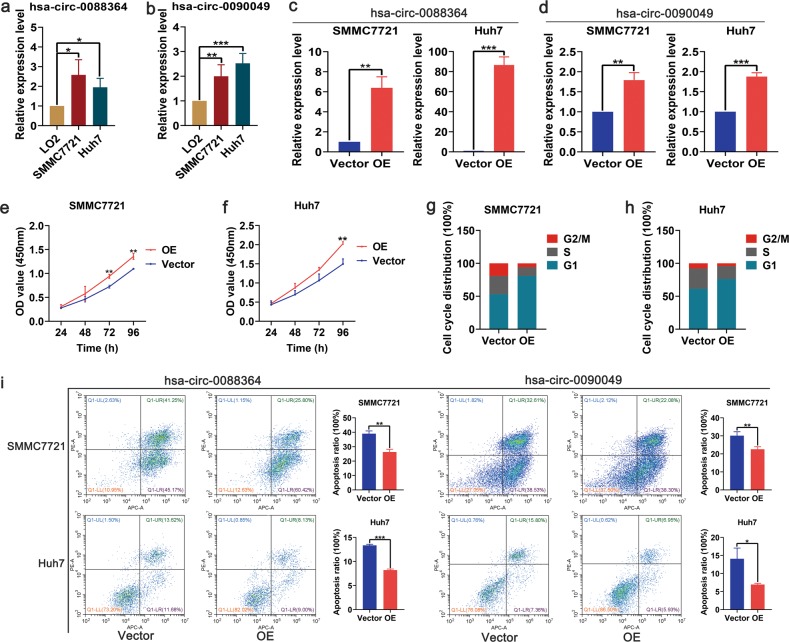
Biological effects of hsa_circ_0088364 and hsa_circ_0090049 on cell proliferation, cell cycle and cell apoptosis in hepatocellular carcinoma (HCC). High expression of hsa_circ_0088364 (**a**) and hsa_circ_0090049 (**b**) in SMMC7721 and Huh7 cells compared to in hepatocyte LO2. Overexpression of hsa_circ_0088364 (**c**) and hsa_circ_0090049 (**d**) in SMMC7721 and Huh7 with lentivirus. Overexpressed hsa_circ_0090049 promotes proliferation of SMMC7721 (**e**) and Huh7 (**f**). Overexpressed hsa_circ_0090049 promotes SMMC7721 (**g**) and Huh7 (**h**) cells into G1 phase. Overexpressed hsa_circ_0088364 and hsa_circ_0090049 inhibits NC-induced apoptosis. OE overexpression

**Fig. 5:**
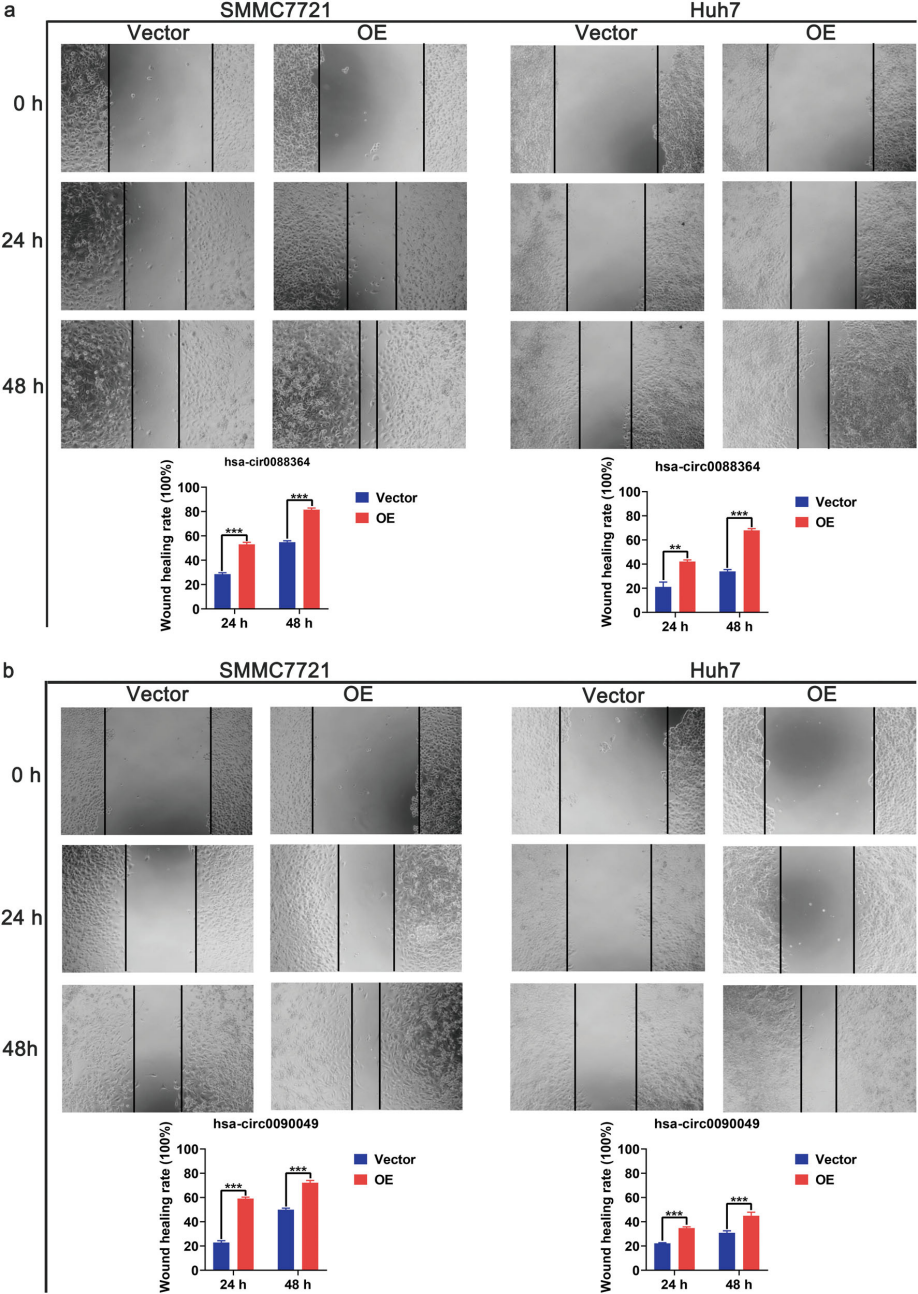
Biological effects of hsa_circ_0088364 and hsa_circ_0090049 on cell migration in hepatocellular carcinoma. Overexpressed hsa_circ_0088364 (**a**) and hsa_circ_0090049 (**b**) promote migration of SMMC7721 and Huh7. OE overexpression

Since hsa_circ_0088364 and hsa_circ_0090049 may be therapeutic targets of NC, we focused on their biological effects in HCC. We firstly found that the expression of hsa_circ_0088364 and hsa_circ_0090049 was higher in SMMC7721 and Huh7 cells than in normal hepatocytes LO2 (Fig. 4a, b), suggesting that they may act as tumour promoting circRNAs. Then, we overexpressed the two circRNAs in SMMC7721 and Huh7 with lentivirus (Fig. 4c, d) for in vitro experiments. The CCK8 assay showed that hsa_circ_0090049 promoted the proliferation of SMMC7721 and Huh7 cells (Fig. 4e, f), however, hsa_circ_0088364 had no effect on the proliferation of HCC cells (Supplementary Fig. S1a, b). Consistent with this, hsa_circ_0090049 could promote HCC cells into the G1 phase (Fig. 4g, h), while hsa_circ_0088364 has no effect on the cell cycle (Supplementary Fig. S1c, d). Subsequently, we evaluated the effects of hsa_circ_0088364 and hsa_circ_0090049 on NC-induced apoptosis of HCC cells by treating SMMC7721 and Huh7 cells with 3 μmol/L and 10 μmol/L NC for 24 h, respectively. We found that high expression of hsa_circ_0088364 and hsa_circ_0090049 inhibited NC-induced apoptosis (Fig. 4i). In addition, we used a wound-healing assay to explore whether hsa_circ_0088364 and hsa_circ_0090049 affect HCC cell migration. The results showed that both hsa_circ_0088364 and hsa_circ_0090049 promoted the migration of SMMC7721 and Huh7 cells (Fig. 5).

### Identification of circRNA-miRNA and miRNA-mRNA interactions

**Fig. 6:**
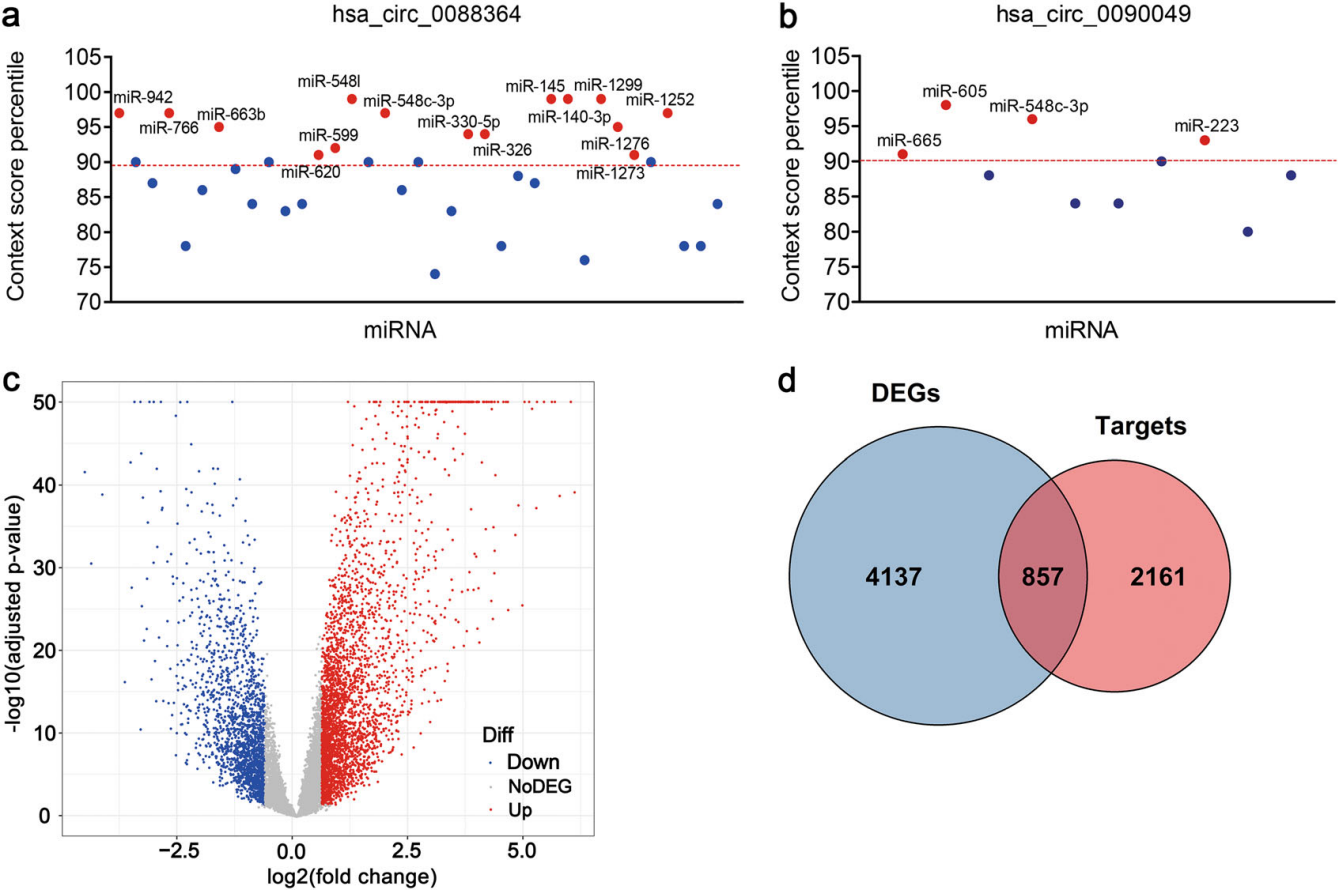
Identification of circRNA-miRNA and miRNA-mRNA interactions. Context score percentile of miRNAs that bind to hsa_circ_0088364 (**a**) and hsa_circ_0090049 (**b**). miRNAs with a context score percentile >90 are indicated by red dots. **c** Volcano plot showing differently expressed genes (DEGs) in hepatocellular carcinoma from The Cancer Genome Atlas. **d** Overlapping genes between DEGs and validated miRNA target genes

An increasing number of studies have reported that circRNAs function as miRNA sponges to exert their roles in many human diseases, including malignant tumours. Here, we predicted miRNA binding sites of hsa_circ_0088364 and hsa_circ_0090049 based on the CircInteractome. In total, 19 circRNA-miRNA interactions consisting of two circRNAs (hsa_circ_0088364 and hsa_circ_0090049) and 18 miRNAs were identified (Table 1). Of the 18 miRNAs, four bound to hsa_circ_0090049, 15 bound to hsa_circ_0088364, and has-miR-548c-3p bound to both hsa_circ_0088364 and hsa_circ_0090049 (Fig. 6a, b).

The following criteria were used in the analysis: |log2 (fold change)| >0.585 and adjusted *p*-value < 0.05. With these criteria, 4995 DEGs including 3154 upregulated genes and 1841 downregulated genes in HCC were obtained from The Cancer Genome Atlas (TCGA) (Fig. 6c). Simultaneously, 3018 experimentally validated target mRNAs of 17 miRNAs other than miR-1273 were obtained from the miRWalk and were intersected with the 4995 DEGs. Finally, 857 miRNA-associated DEGs in HCC were determined (Fig. 6d).

### Detection of clinically significant gene co-expression modules

**Fig. 7:**
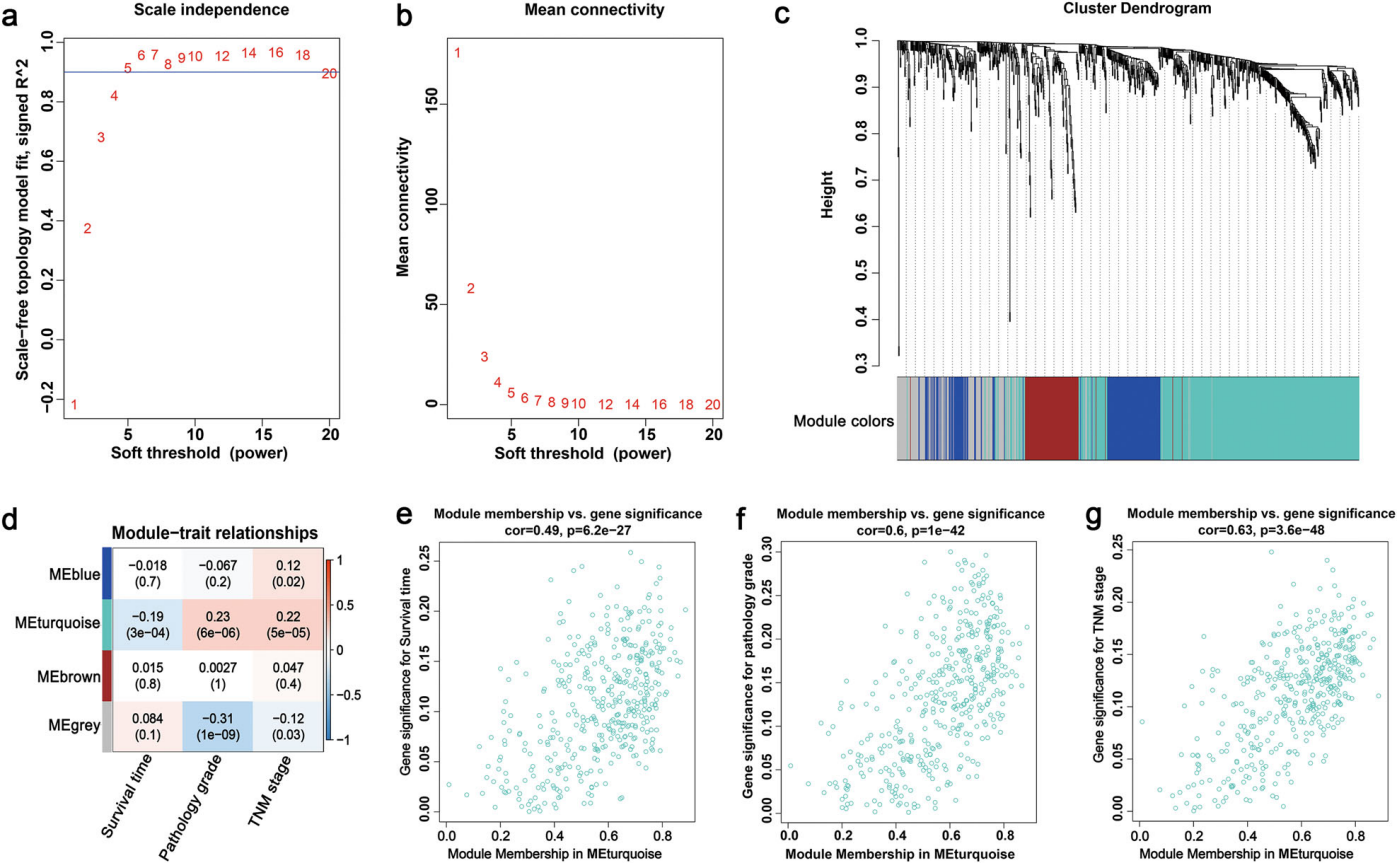
Identification of modules associated with clinical traits of hepatocellular carcinoma (HCC) by using the weighted gene co-expression network analysis. **a** Analysis of the scale-free topology fit index for various soft-threshold powers. **b** Analysis of the mean connectivity for various soft-threshold powers. **c** Clustering dendrogram showing modules identified based on the soft-threshold power of five. **d** Heatmap of correlations between module eigengenes and clinical traits (survival time, pathology grade and TNM stage) of HCC. The module-trait relationships were showed by correlation coefficient values and *p*-values (in parenthesis). Relationships between genes in module turquoise and genes significance for survival (**e**), grade (**f**) and TNM stage (**g**) were investigated by Pearson correlation

A WGCNA analysis was performed based on the 857 miRNA-associated DEGs in HCC to identify gene co-expression modules that were correlated with the clinical outcome of patients with HCC. Based on a soft-threshold power of five that enabled a scale-free topology fit index above 0.9 (Fig. 7a, b), three modules with genes ranging from 102 to 423 were identified (Fig. 7c), among which Module Eigengene turquoise (MEturquoise) with 423 genes was significantly correlated with the survival time, pathology grade and TNM stage in patients with HCC (*p*-value < 0.05; Fig. 7d). Furthermore, the module memberships in MEturquoise were significantly correlated with the genes that were significant for survival, pathology grade and TNM stage (Fig. 7e, f). Thus, our subsequent study focused on MEturquoise since it was clinically significant.

### Construction of circRNA-miRNA-mRNA network

**Fig. 8:**
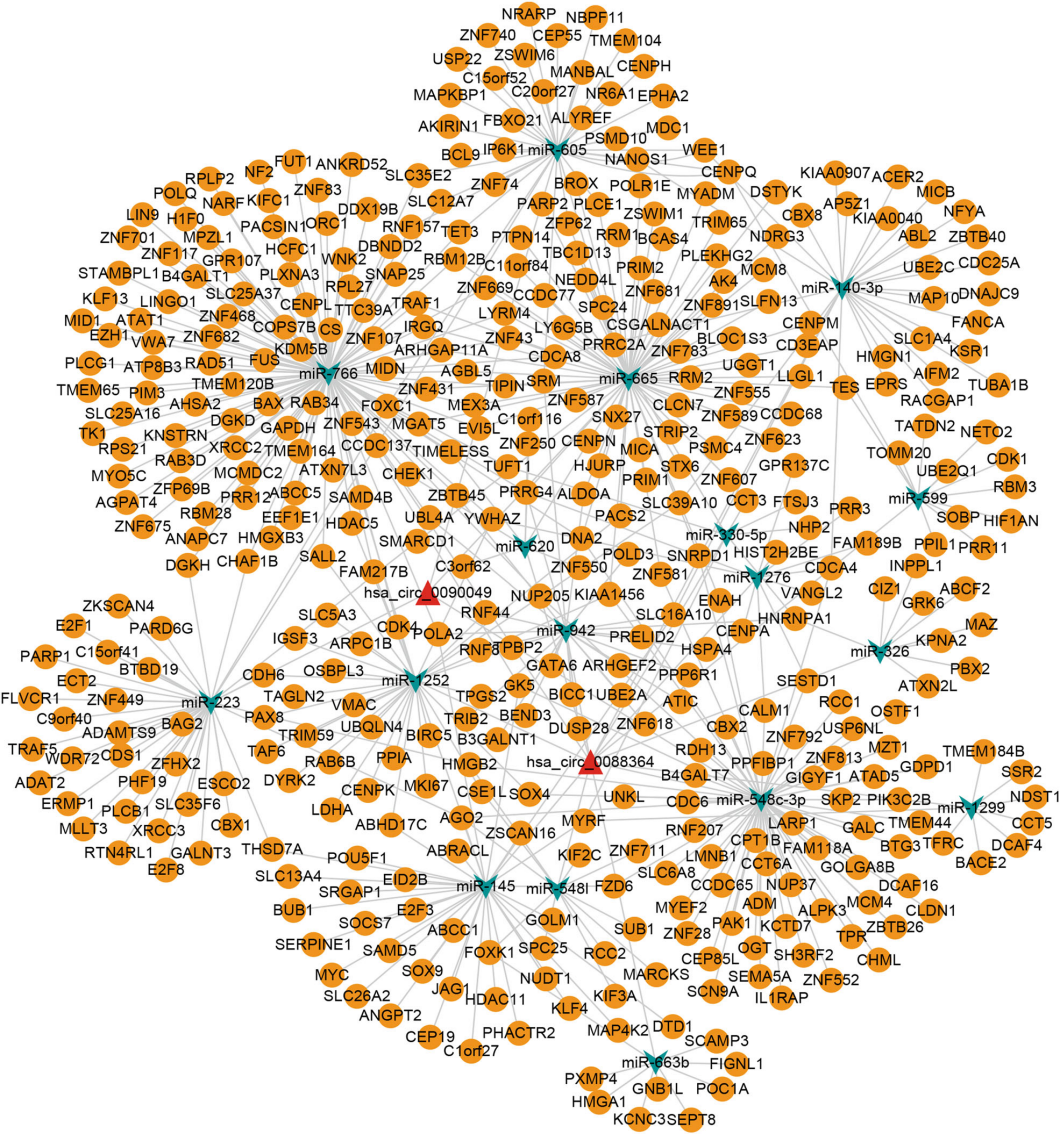
A circRNA-miRNA-mRNA regulatory network. The network consisting of two circRNAs (hsa_circ_0088364 and hsa_circ_0090049), 17 miRNAs and 423 mRNAs was generated by Cytoscape 3.6.1

We selected 423 genes in MEturquoise for circRNA-miRNA-mRNA network construction. Following integration of circRNA-miRNA interactions and miRNA-mRNA interactions, a network consisting of two circRNA (hsa_circ_0088364 and hsa_circ_0090049), 17 miRNAs and 423 mRNAs was constructed (Fig. 8).

### GO annotations and KEGG pathway analyses of MEturquoise

**Fig. 9:**
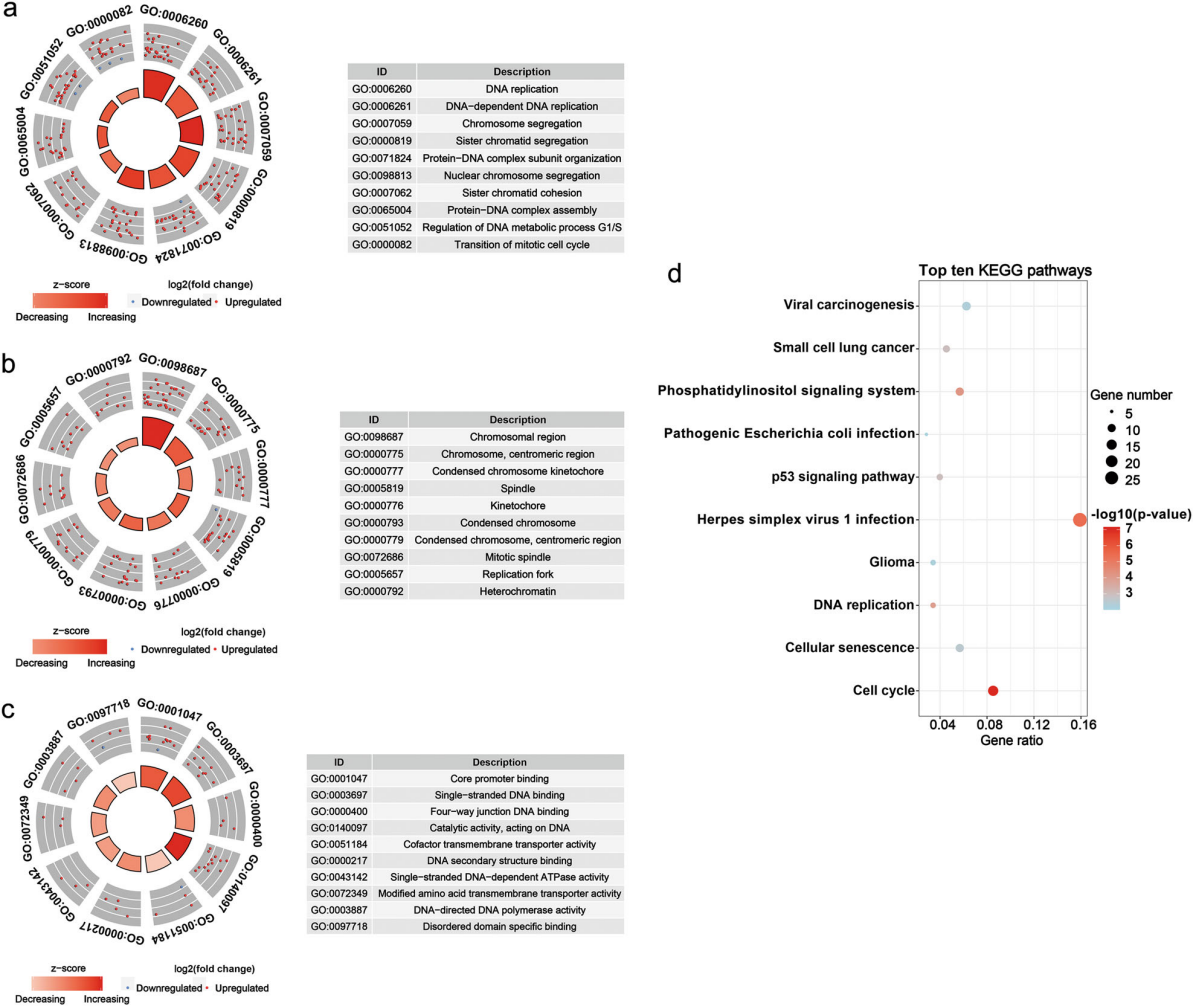
TOP ten Gene Ontology (GO) functional annotations and Kyoto Encyclopedia of Genes and Genomes (KEGG) pathways of the 423 genes in MEturquoise. **a** Biological processes, **b** cellular components, **c** molecular functions and **d** KEGG pathways

The 423 genes in MEturquoise were categorized into three functional groups of biological process (BP), cellular component (CC) and molecular function (MF). The top ten GO annotations are shown in Fig. 9a–c. The most enriched term for the 423 genes in BP was “DNA replication”, that in CC was “chromosomal region” and that in MF was “core promoter binding”. KEGG pathway analysis was also conducted based on the 423 genes. The top ten KEGG pathways are shown in Fig. 9d, some of which were tumour-related signalling cascades, such as “cell cycle” and “p53 signalling pathway”.

### Identification and validation of survival-related hubgenes in MEturquoise

**Fig. 10:**
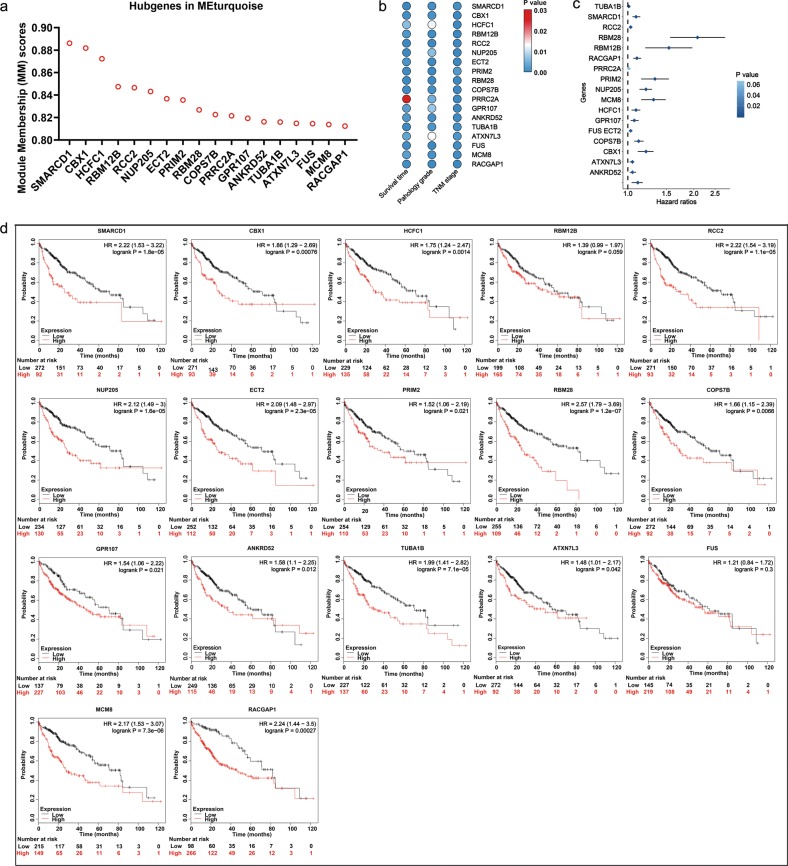
Identification of 18 hubgenes (SMARCD1, CBX1, HCFC1, RBM12B, RCC2, NUP205, ECT2, PRIM2, RBM28, COPS7B, PRRC2A, GPR107, ANKRD52, TUBA1B, ATXN7L3, FUS, MCM8 and RACGAP1) in MEturquoise. **a** Module Member scores of the 18 hubgenes in MEturquoise. **b** Relationships between the 18 hubgenes and the survival time, pathology grade and TNM stage of patients with hepatocellular carcinoma (HCC). **c** Forest plot revealing the prognostic value of the hubgenes in HCC based on data from The Cancer Genome Atlas. **d** Relationships between the hubgenes expression and the overall survival of HCC patients by data from the Kaplan–Meier plotter

For the 423 genes in MEturquoise, we determined 18 genes as hubgenes (SMARCD1, CBX1, HCFC1, RBM12B, RCC2, NUP205, ECT2, PRIM2, RBM28, COPS7B, PRRC2A, GPR107, ANKRD52, TUBA1B, ATXN7L3, FUS, MCM8 and RACGAP1), which were highly associated with MEturquoise with Module Membership (MM) scores > 0.8 (Fig. 10a) and were remarkably correlated with the survival time, pathology grade and TNM stage in patients with HCC (Fig. 10b). All of the 18 genes were upregulated in HCC based on data from the TCGA (Supplementary Fig. S2). Univariate Cox analysis revealed that the high expression of the hubgenes in addition to PRRC2A indicated a poor overall survival (OS) in patients with HCC. The relationships between the hubgenes expression and the OS of HCC patients were corroborated by 364 RNA-seq data from the Kaplan–Meier plotter (http://kmplot.com/analysis/index.php?p=service&cancer=liver_rnaseq) (Fig. 10d), among which no survival data of PRRC2A was provided. RBM12B and FUS showed no relationships with the OS of the 364 patients with HCC, but the high expression of the remaining 15 genes indicated worse OS. In addition, the associations between these hubgenes expression and the relapse-free survival (RFS) and the progression-free survival (PFS) of HCC patients were also verified by 316 and 370 RNA-seq data from the Kaplan–Meier plotter, respectively (Supplementary Fig. S3, S4). The expression levels of the proteins encoded by the 18 genes in HCC and normal liver tissues are shown in Supplementary Fig. S5 based on the data from The Human Proteins Atlas (http://www.proteinatlas.org/about), among which no protein expression information of HCFC1, NUP205, ECT2, PRIM2, MCM8 were provided.

## Discussion

In this study, we identified two circRNAs (hsa_circ_0088364 and hsa_circ_0090049) that could be potential therapeutic targets of NC in HCC and revealed the potential mode of action of the circRNA-miRNA-mRNA network in HCC by employing a combination strategy of RNA-seq, RT-qPCR, in vitro experiments and WGCNA analysis. Our findings, for the first time, provide novel insights into the potential of circRNAs as therapeutic targets of NC against HCC.

NC is a natural bioactive compound that has various biological properties, including antiparasitic, antimicrobial, antioxidant and anti-inflammatory activities^14–16^. Recently, increasing numbers of studies have proposed that NC can be used as an antitumour agent due to its inhibitory effect on tumour development. A study conducted by Liu et al.^17^ showed that NC inhibited the malignant biological behavior of human glioblastoma in vitro. Sun et al.^18^ revealed the inhibitory effect of NC on cell migration and invasion of ovarian cancer cells. Kim et al.^19^ proposed that NC repressed human oral tumour growth in vitro and in vivo. In this study, we demonstrated the antitumour role of NC in HCC, which was consistent with the previous studies conducted by Liao et al.^7^, Lin et al.^5^ and Ou et al.^6^. Our research group has long focused on the anti-tumour effect of NC on HCC and have published several articles^8,13^, and these studies revealed that NC was a promising chemotherapeutic agent against HCC. However, more experiments are needed to gain insights into the anti-HCC mechanism of NC.

With the development of high-throughput RNA-seq technology, circRNAs have received increasing attention. The close links between circRNA changes and human malignant tumours, including HCC, have been reported by multiple studies^20–23^. Zhang et al.^24^ found that circRNA_104075 stimulated YAP-dependent tumourigenesis by the regulation of HNF4a in HCC. Yu et al.^25^ demonstrated that circRNA cSMARCA5 repressed tumour growth and metastasis in HCC. Yao et al.^26^ highlighted that hsa_circ_0016788 was upregulated in HCC. Knockdown of hsa_circ_0016788 inhibited the malignant biological behaviours of HCC cells in vitro and decrease tumour growth in vivo. These studies indicated the important roles of circRNAs in the initiation and progression of HCC, which provided novel insights into their application as therapeutic targets in HCC.

**Fig. 11:**
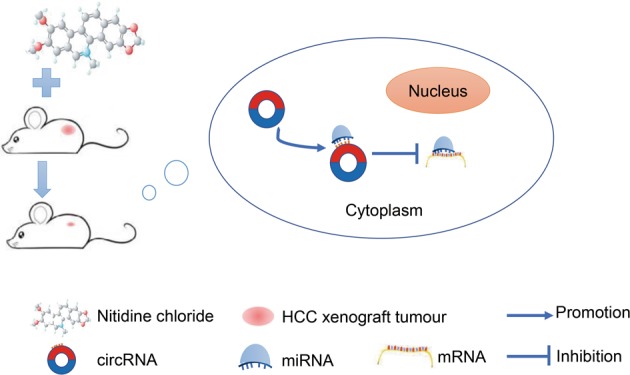
Underlying action mode of the circRNA-miRNA-mRNA network in nitidine chloride anti-hepatocarcinoma. HCC hepatocellular carcinoma

Here, a circRNA sequencing analysis was firstly conducted based on three pairs of NC-treated and NC-untreated HCC xenograft tumour tissues. A total of 297 differently expressed circRNAs were observed between the two groups. Following RT-qPCR validation, we selected hsa_circ_0088364 and hsa_circ_0090049 for further study. The in vitro experiments showed that these two circRNAs inhibited the malignant biological behavior of HCC, suggesting that they may play important roles in the development of HCC. However, the molecular mechanism by which these two circRNAs promote HCC progression remains unknown. Growing evidence has shown that circRNAs contain a large amount of miRNA binding sites and play key roles as “miRNA sponges” in the pathogenesis and progression of human tumours. For example, cirRNA CDR1as contains more than 70 miRNA binding sites and acts as an miR-7 sponge in cancer^27^. CircRNA NF1 functions as a miRNA sponge, accelerating gastric cancer progression by absorbing miR-16^10^. CircRNA circMTO1 represses bladder cancer metastasis by sequestering miR-21^28^. We have identified a circRNA-miRNA-mRNA network that may contribute to the initiation and progression of HCC in our previous study^12^. Here, we aimed to further investigate the role of the circRNA-miRNA-mRNA axis in the treatment of HCC with NC. By using a computational biology analysis, 19 circRNA-miRNA interactions including two circRNAs (hsa_circ_0088364 and hsa_circ_0090049) and 18 miRNAs were identified. We then collected target mRNAs of the 18 miRNAs and intersected them with 4995 DEGs in HCC, obtaining 857 miRNA-associated DEGs. WGCNA analysis was performed based on the 857 genes to identify gene co-expression networks and their underlying clinical significances. WGCNA is a system-level method for identifying modules of highly correlated genes from microarray or RNA-seq datasets and for relating modules to clinical traits^29^, which has been applied in various biological contexts, including cancer^30,31^. In this study, we identified three modules of the 857 genes by using the WGCNA approach, among which MEturquoise with 423 genes showed close links with the survival time, pathology grade and TNM stage of patients with HCC. A circRNA-miRNA-mRNA network containing two circRNAs (hsa_circ_0088364 and hsa_circ_0090049), 17 miRNAs and 423 genes was then constructed. To further elucidate the underlying biological function and action mechanism of the aforementioned regulatory network in HCC, we performed functional annotation and pathway enrichment analyses based on the 423 genes in MEturquoise. The results of the GO and KEGG analyses revealed that these genes were involved in DNA replication- and cell cycle-related biological processes and signalling cascades, suggesting their pivotal roles in HCC^32^. Additionally, we found that the 423 genes also participated in some other tumour-related pathways, such as “p53 signalling pathway” and “Viral carcinogenesis”. All of these findings indicated that the identified circRNA-miRNA-mRNA network may play important role in the occurrence and progression of HCC and this network and could be targets of NC against HCC (Fig. 11).

Since MEturquoise was a clinically significant module, we determined the highly connected intra-modular genes in MEturquoise. With the MM > 0.8, 18 hubgenes (SMARCD1, CBX1, HCFC1, RBM12B, RCC2, NUP205, ECT2, PRIM2, RBM28, COPS7B, PRRC2A, GPR107, ANKRD52, TUBA1B, ATXN7L3, FUS, MCM8 and RACGAP1) were identified. All of the 18 genes were upregulated and were negatively correlated with the survival time and positively correlated with the pathology grade and TNM stage of HCC patients. Additionally, their relationships with OS, RFS and PFS of HCC patients were verified by data from the Kaplan–Meier plotter, indicating that these genes may be biomarkers for clinical outcome prediction in patients with HCC. Since few studies have previously reported the relationships between the 18 genes and HCC, further experiments are necessary to validate our findings.

In conclusion, in this study, we identified a population of circRNAs differentially expressed in NC-treated and NC-untreated HCC xenografts in nude mice tissues by high-throughput sequencing analysis and verified the expression of hsa_circ_0088364 and hsa_circ_0090049 by RT-qPCR. Following in vitro experiments, we found that these two circRNAs play a role in the progression of HCC. Our findings provide interesting potential clues into the possibilities of circRNAs as therapeutic targets of NC in HCC. Nevertheless, we have not verified the biological function of the two circRNAs in HCC in vivo, nor have we determined how NC targets the two circRNA against HCC. Therefore, more rigorous experiments are still needed, and we will continue to focus on this issue in our further studies.

## Materials and methods

### Drug preparation

NC (96% purity) was purchased from Chengdu Herbpurify Co., LTD. (Chengdu, China). We dissolved NC in dimethyl sulfoxide (DMSO) and diluted it to the working concentration with medium.

### Animals and treatments

All animal experiments were performed according to the Guide for the Care and Use of Laboratory Animals (the Shanghai SLAC Laboratory Animal of China, 2015) and were approved by the Ethics Committee of the First Affiliated Hospital of Guangxi Medical University (Nanning, China). BALB/c nude mice were purchased from Guangxi Medical University Animal Center (Guangxi, China). The mice were housed in pathogen-free conditions with a 12 h light/12 h dark cycle at room temperature. SMMC7721 cells (1 × 10^7^ cells/L) were injected into the right armpit of each mouse. Fourteen days later, mice with a tumour volume of approximately 70 mm^3^ were randomized into two groups: The negative control group was intraperitoneally injected with saline, and the NC group was intraperitoneally injected with 7 mg/kg/d NC. After 15 days of drug administration every other day, the mice were euthanized and the tumour tissues were excised and stored at −80 ˚C.

### High-throughput circRNA sequencing

Total RNA was extracted with TRIzol Regent (Invitrogen, USA). RNA degradation and contamination were detected by using 1% agarose gels. RNA purity and integrity were checked by using the NanoPhotometer®spectrophotometer (IMPLEN, CA, USA) and the RNA Nano 6000 Assay Kit of the Agilent Bioanalyzer 2100 system (Agilent Technologies, CA, USA), respectively.

The sequencing library was generated by NEBNext® Ultra™ Directional RNA Library Prep Kit for Illumina® (NEB, USA) following the manufacturer’s protocols and the library quality was monitored on the Agilent Bioanalyzer 2100 system. Fragments were sequenced on the HiSeq2000 platform by using 150 bp paired-end reads. Raw reads with adaptors, >5% unknown nucleotides and low-quality bases were removed. Qualified reads were mapped against human genome references (GRCh37/hg19) by using bowtie2^33^ and BWA^34^. Unmapped reads were extracted and realigned to genome references to identify candidate back-spliced junction reads. The expression level of each circRNA was determined by the back-spliced reads per million mapped reads (RPM).

### Differently expressed circRNAs

The *DESeq2* package in Bioconductor was used to determine differently expressed circRNAs, according to the criteria of |log2 (fold change) | >1 and *p*-value < 0.05.

### RT-qPCR

Total RNA was isolated from cells and tissues with NucleoZol (MACHEREY-NAGAL, Düren, Germany). cDNA reverse-transcription was conducted with the PrimeScript^TM^RT reagent Kit (TaKaRa Biotechnology (Dalian) Co., Ltd., Dalian, China). RT-qPCR was conducted with an ABI 7300 PCR system (Applied Biosystems, CA, USA) for 40 cycles by using SYBR^®^ Premix Ex Taq™ II (TaKaRa Biotechnology (Dalian) Co., Ltd.). The primer sequences of hsa_circ_0088364 (forward 5′-TTAGTATTTGGAGCAGACCCTC-3′; reverse 5′-ACCACAGCAATCTTGTCAAGG-3′) and hsa_circ_0090049 (forward 5′-TATCGCCAGTCCAGCAGC-3′; reverse 5′-GCTATCCCATGTCCAATTTCAT-3′) were synthesized by GenePharma (Shanghai, China) and GeneSeed (Guangzhou, China). CircRNA expression was determined by using the formula 2^-△CT^ in tumour tissues and 2^-△△CT^ in cells with ACTB (TaKaRa Biotechnology) as the internal control.

### Cell culture

The human SMMC7721 and Huh7 cells were cultured in DMEM medium supplemented with 10% fetal bovine serum (Lonsa Science SRL, Montevideo, Uruguay) in an atmosphere of 5% CO_2_ at 37 °C.

### **C**ell transfection

Full length cDNA of hsa_circ_0090049 and hsa_circ_0088364 were amplified and cloned into GV502 vector. Mock plasmid without hsa_circ_0090049 and hsa_circ_0088364 cDNA was served as a control vector. All of the lentiviral vectors were transfected into SMMC7721 and Huh7 cells following the manufacturer’s instructions. Further experiments were conducted at 72 h post-transfection.

### CCK8 assay

The inhibition effects of NC on SMMC7721 and Huh7 cells were determined by using a CCK8 assay. Cells were seeded in 96-well plates at a density of 5000 cells/well for 24 h and were then treated with various concentrations of NC and DMSO. After the cells were exposed to NC for 24, 48 and 72 h, the medium was added with CCK8 reagent and the optical density (OD) value of each well was measured. The cell inhibition rate was calculated as: [(OD_negative control group_− OD_NC-treated group_)/(OD_negative control group_− OD_blank group_)]×100%.

The CCK8 assay was also used to detect the effect of hsa_circ_0090049 and hsa_circ_0088364 on cell viability. Cells transfected with lentivirus vectors were seeded in 96-well plates (4000 cells/well) for 24, 48, 72 and 96 h. Cell viability was determined by detecting OD values.

### Cell cycle analysis

Cells transfected with lentivirus vectors or treated with NC were harvested for cell cycle analysis by flow cytometry with propidium iodide (PI) staining according to the manufacturer’s instructions. Cell cycle distribution was analyzed by using CytoFLEX (Beckman Coulter, Indianapolis, USA).

### Cell apoptosis analysis

Cell apoptosis induced by NC was detected by flow cytometry with Annexin V-APC/PI double staining kit (BestBio, Shanghai, China) following the manufacture’s protocols. The percentage of early apoptotic cells (APC+/PI−) and late apoptotic cells (APC+/PI+) were calculated by using CytoFLEX (Beckman Coulter).

### Wound-healing assay

Cells were cultured in 96-well plates for overnight. A micropipette tip was used to create a scratch in each well when the cells were completely confluent. Photographs of the wound were taken at 0, 24 and 48 h. Wound width was calculated by using ImageJ (National Institutes of Health, New York, USA).

### Identification of circRNA-miRNA interactions

CircRNA-miRNA interactions were predicted by the CircInteractome^35^. miRNAs with the context score percentile >90 were selected^36^.

### Collection of miRNA target genes

Validated miRNA-target interactions that were supported by published literatures were collected from the *Validated Target Module* of the miRwalk^37^.

### Obtaining differently expressed genes in HCC

RNA-seq data of 371 HCC and 50 normal liver samples were downloaded from the TCGA database^38^. The *DESeq2* package in R were used to screen DEGs

### WGCNA

The *WGCNA* package in R was used to construct the weighted gene co-expression network. Before module detection, the gene expression matrix was imported into the R version 3.5.1 (http://mirror.lzu.edu.cn/CRAN/). We calculated the strength of the Pearson connection between each gene pair, obtaining an adjacency matrix by raising the matrix to a soft-threshold power by using the *pickSoftThreshold* function of *WGCNA*. This function provides a scale-free topology fit index, which reaches values above 0.9 for low powers (<30), meaning that the topology of the network is scale-free^39^. With the selected power value, we identified network modules with the *minModuleSize* of 30 and the *mergeCutHeight* of 0.25. Each module was assigned a specific colour, and a grey colour indicates that the genes are outside of any module. The clinical data including survival time, pathology grade and TNM stage of 371 HCC samples were obtained from the TCGA. We then calculated the correlation between each module and the clinical traits to find clinically significant modules.

### GO and KEGG pathway analyses

GO functional annotations and KEGG pathway enrichment analyses were performed by the *clusterProfiler* package in Bioconductor.

### Construction of the circRNA-miRNA-mRNA network

The circRNA-miRNA and miRNA-mRNA interactions were combined for the construction of the circRNA-miRNA-mRNA regulatory network by using Cytoscape 3.6.1^40^.

### Identification of hubgenes

Highly connected intra-modular genes with high MM scores are considered as hubgenes in a given module. The MM value of each gene was measured by using the *signedKME* function of *WGCNA*.

### Statistical analysis

All of the experiments were conducted in triplicate. Differences between two groups were analyzed with Student’s *t*-test by using SPSS 22.0 (IBM, New York, USA), and a p-value < 0.05 indicated a statistically significant difference.

## Supplementary information

Supplementary figures.

Supplementary figure legends.
