# Supplementary material for: High throughput circRNA sequencing analysis reveals novel insights into the mechanism of nitidine chloride against hepatocellular carcinoma
Source: Cell Death Dis. 2019 Sep 10;10(9):658. doi: 10.1038/s41419-019-1890-9 (PMC6737102; doi:10.1038/s41419-019-1890-9)

High throughput circRNA sequencing analysis reveals novel insights into the mechanism of nitidine chloride against hepatocellular carcinoma

Running title: circRNA as therapeutic target of nitidine chloride in hepatocellular carcinoma

Dan-dan Xiong[1*], Zhen-bo Feng[1*], Ze-feng Lai[2], Yue Qin[2], Li-min Liu[3], Hao-xuan Fu[2], Rong-quan He[4], Hua-yu Wu[5], Yi-wu Dang[1#], Gang Chen[1#], Dian-zhong Luo[1]

[1]Department of Pathology, First Affiliated Hospital of Guangxi Medical University, Nanning, China

[2]Pharmaceutical College, Guangxi Medical University, Nanning, China

[3]Department of Toxicology, Pharmaceutical College, Guangxi Medical University, Nanning, China

[4]Department of Medical Oncology, First Affiliated Hospital of Guangxi Medical University, Nanning, China

[5]Department of Cell Biology & Genetics, School of Preclinical Medicine, Guangxi Medical University, Nanning, China

Corresponding authors:

Professor Dr. Gang Chen, Department of Pathology, First Affiliated Hospital of Guangxi Medical University, No. 6. Shuangyong Rd., Nanning, Guangxi Zhuang Autonomous Region, 530021, P.R. China. Email: chengang@gxmu.edu.cn. Tel: +8615277192143

Yi-wu Dang, Department of Pathology, First Affiliated Hospital of Guangxi Medical University, No. 6. Shuangyong Rd., Nanning, Guangxi Zhuang Autonomous Region, 530021, P.R. China. Email: dangyiwu@126.com. Tel: +8613737034122

[*] These authors contributed equally to this work

Supplementary Fig S1

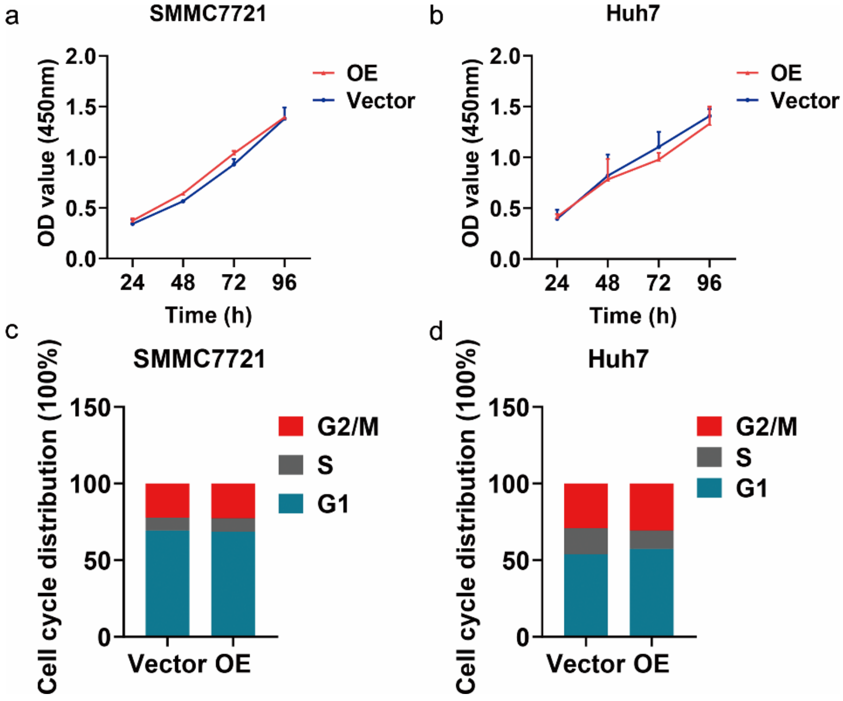

Supplementary Fig S2

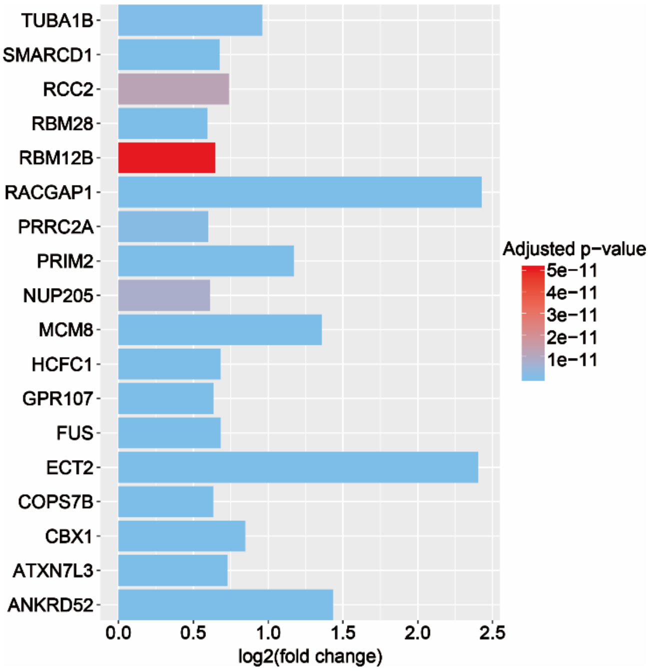

Supplementary Fig S3

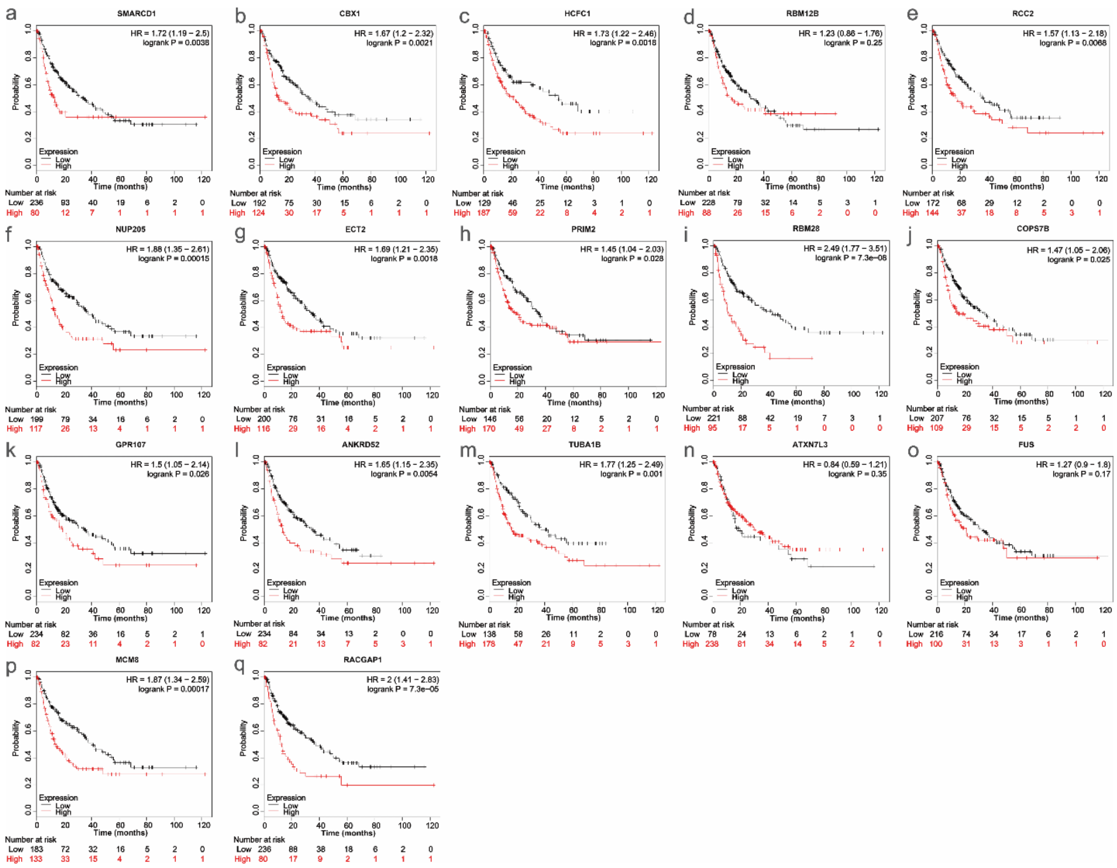

Supplementary Fig S4

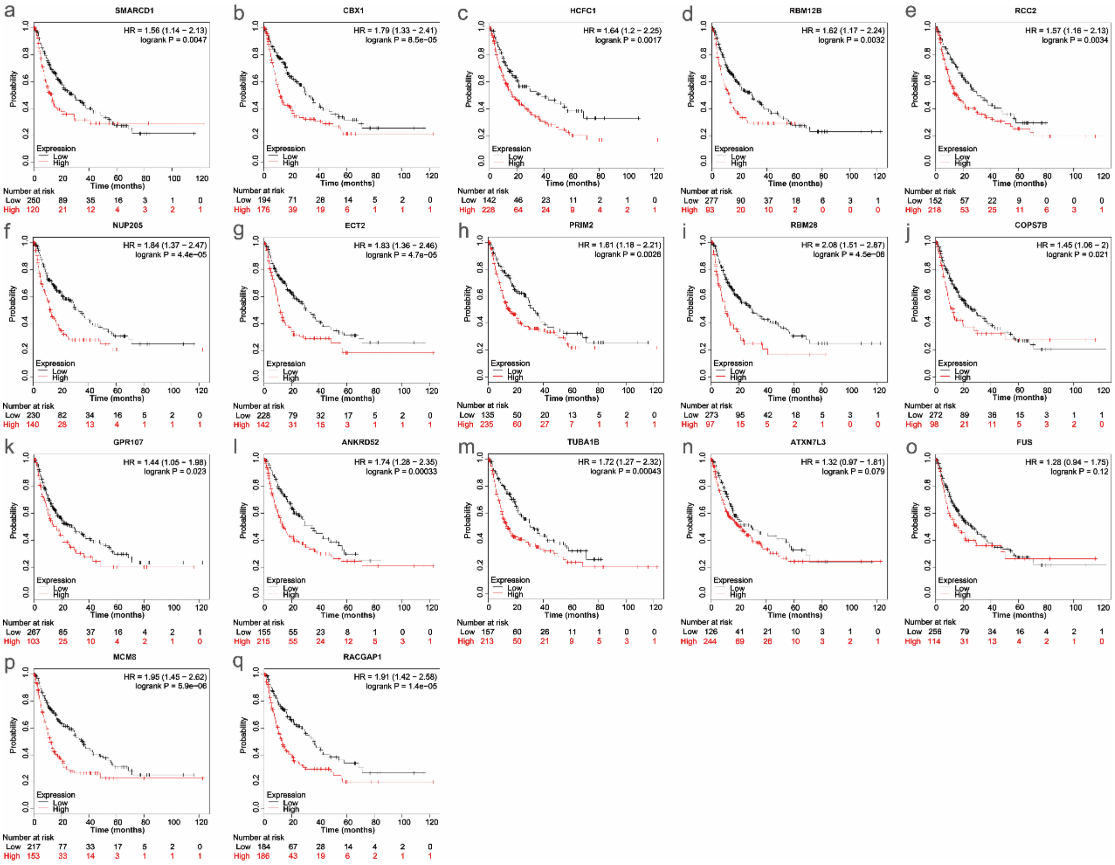

Supplementary Fig S5

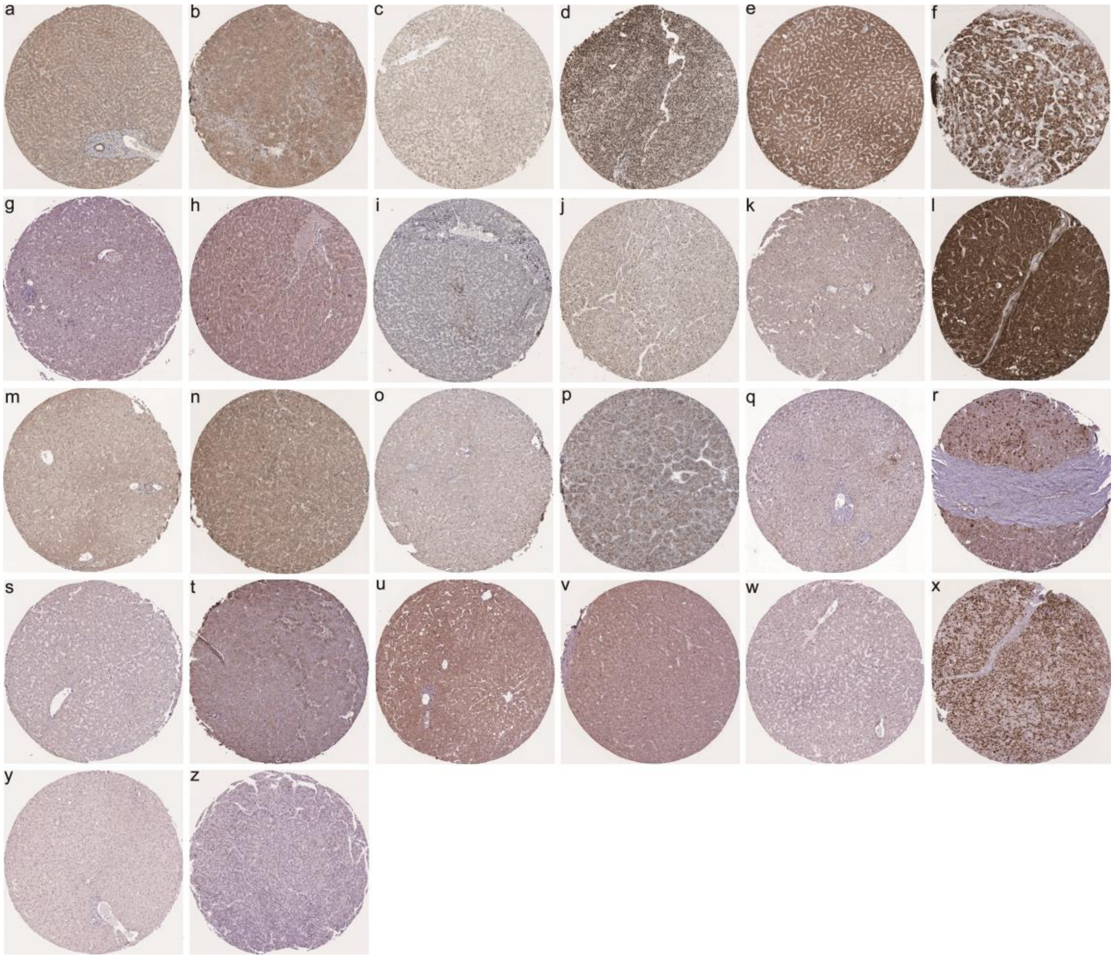

Supplement: Supplementary file 1 — Supplementary figures. [file 41419_2019_1890_MOESM1_ESM.pdf]
