# Supplementary material for: High throughput circRNA sequencing analysis reveals novel insights into the mechanism of nitidine chloride against hepatocellular carcinoma
Source: Cell Death Dis. 2019 Sep 10;10(9):658. doi: 10.1038/s41419-019-1890-9 (PMC6737102; doi:10.1038/s41419-019-1890-9)
Supplement: Supplementary file 2 — Supplementary figure legends. [file 41419_2019_1890_MOESM2_ESM.docx]

**High throughput circRNA sequencing analysis reveals novel insights into the mechanism of nitidine chloride against hepatocellular carcinoma**

**Running title: circRNA as therapeutic target of nitidine chloride in hepatocellular carcinoma**

Dan-dan Xiong^1*^, Zhen-bo Feng^1*^, Ze-feng Lai^2^, Yue Qin^2^, Li-min Liu^3^, Hao-xuan Fu^2^, Rong-quan He^4^, Hua-yu Wu^5^, Yi-wu Dang^1#^, Gang Chen^1#^, Dian-zhong Luo^1^

^1^Department of Pathology, First Affiliated Hospital of Guangxi Medical University, Nanning, China

^2^Pharmaceutical College, Guangxi Medical University, Nanning, China

^3^Department of Toxicology, Pharmaceutical College, Guangxi Medical University, Nanning, China

^4^Department of Medical Oncology, First Affiliated Hospital of Guangxi Medical University, Nanning, China

^5^Department of Cell Biology & Genetics, School of Preclinical Medicine, Guangxi Medical University, Nanning, China

**Corresponding authors:**

Professor Dr. Gang Chen, Department of Pathology, First Affiliated Hospital of Guangxi Medical University, No. 6. Shuangyong Rd., Nanning, Guangxi Zhuang Autonomous Region, 530021, P.R. China. Email: [chengang@gxmu.edu.cn](mailto:chengang@gxmu.edu.cn). Tel: +8615277192143

Yi-wu Dang, Department of Pathology, First Affiliated Hospital of Guangxi Medical University, No. 6. Shuangyong Rd., Nanning, Guangxi Zhuang Autonomous Region, 530021, P.R. China. Email: dangyiwu@126.com. Tel: +8613737034122

^*^ These authors contributed equally to this work

**Supplementary Files**

**Supplementary Fig. S1. Effects of hsa-circ-0088364 on cell proliferation (a-b) and cell cycle (c-d) of SMC7721 and Huh7.** OE: overexpression.

**Supplementary Fig. S2. Bar plot showing the expression of 18 hubgenes in hepatocellular carcinoma.**

**Supplementary Fig. S3. Relationships between SMARCD1 (a), CBX1 (b), HCFC1 (c), RBM12B (d), RCC2(e), NUP205 (f), ECT2 (g), PRIM2 (h), RBM28 (i), COPS7B (j), GPR107 (k), ANKRD52 (l), TUBA1B (m), ATXN7L3 (n), FUS (o), MCM8 (p) and RACGAP1 (q) expression and the relapse-free survival of HCC patients by data from the Kaplan-Meier plotter.**

**Supplementary Fig. S4. Relationships between SMARCD1 (a), CBX1 (b), HCFC1 (c), RBM12B (d), RCC2(e), NUP205 (f), ECT2 (g), PRIM2 (h), RBM28 (i), COPS7B (j), GPR107 (k), ANKRD52 (l), TUBA1B (m), ATXN7L3 (n), FUS (o), MCM8 (p) and RACGAP1 (q) expression and the progression-free survival of HCC patients by data from the Kaplan-Meier plotter.**

**Supplementary Fig. S5. Immunohistochemistry staining of proteins encoded by the 18 hubgenes from The Human Protein Atlas**. (a) Moderate expression of SMARCD1 in normal liver tissues (https://www.proteinatlas.org/ENSG00000066117-SMARCD1/tissue/liver#img). (b) Moderate expression of SMARCD1 in hepatocellular carcinoma (HCC) tissues (https://www.proteinatlas.org/ENSG00000066117-SMARCD1/pathology/tissue/liver+cancer#img). (c) Low expression of CBX1 in normal liver tissues (https://www.proteinatlas.org/ENSG00000108468-CBX1/tissue/liver#img). (d) High expression of CBX1 in HCC tissues (https://www.proteinatlas.org/ENSG00000108468-CBX1/pathology/tissue/liver+cancer#img) (e) High RBM12B expression in normal liver tissues (https://www tlas..proteinaorg/ENSG00000183808-RBM12B/tissue/liver#img). (f) High RBM12B expression in HCC tissues (https://www.proteinatlas.org/ENSG00000183808-RBM12B/pathology/tissue/liver+cancer#img). (g) Low expression of RCC2 in normal liver tissues (https://www.proteinatlas.org/ENSG00000179051-RCC2/tissue/liver#img). (h) Low expression of RCC2 in HCC tissues (https://www.proteinatlas.org/ENSG00000179051-RCC2/pathology/tissue/liver+cancer#img). (i) Low expression of RBM28 expression in normal liver tissues (https://www.proteinatlas.org/ENSG00000106344-RBM28/tissue/liver#img). (j) High expression of RBM28 in HCC tissues (https://www.proteinatlas.org/ENSG00000106344-RBM28/pathology/tissue/liver+cancer#img). (k) Low expression of COPS7B in normal liver tissues (https://www.proteinatlas.org/ENSG00000144524-COPS7B/tissue/liver#img). (l) High expression of COPS7B in HCC tissues (https://www.proteinatlas.org/ENSG00000144524-COPS7B/pathology/tissue/liver+cancer#img). (m) Low expression of PRRC2A in normal liver tissues (<https://www.proteinatlas.org/ENSG00000204469-PRRC2A/tissue/liver#img). (n>) Moderate expression of PRRC2A in HCC tissues (<https://www.proteinatlas.org/ENSG00000204469-PRRC2A/pathology/tissue/liver+cancer#img>). (o) Low expression of GPR107 in normal liver tissues (<https://www.proteinatlas.org/ENSG00000148358-GPR107/tissue/liver#img>). (p) Moderate expression of GPR107 in HCC tissues (https://www.proteinatlas.org/ENSG00000148358-GPR107/pathology/tissue/liver+cancer#img). (q) ANKRD52 protein was not detected in normal liver tissues (https://www.proteinatlas.org/ENSG00000139645-ANKRD52/tissue/liver#img). (r) Low expression of ANKRD52 in HCC tissues (<https://www.proteinatlas.org/ENSG00000139645-ANKRD52/pathology/tissue/liver+cancer#img>). (s) Low expression of TUBA1B in normal liver tissues (https://www.proteinatlas.org/ENSG00000123416-TUBA1B/tissue/liver#img). (t) Moderate expression of TUBA1B in HCC tissues (<https://www.proteinatlas.org/ENSG00000123416-TUBA1B/pathology/tissue/liver+cancer#img>). (u) Low expression of ATXN7L3 in normal liver tissues (https://www.proteinatlas.org/ENSG00000087152-ATXN7L3/tissue/liver#img). (v) Moderate expression of ATXN7L3 in HCC tissues (<https://www.proteinatlas.org/ENSG00000087152-ATXN7L3/pathology/tissue/liver+cancer#img>). (w) Moderate expression of FUS in normal liver tissues (https://www.proteinatlas.org/ENSG00000089280-FUS/tissue/liver#img). (x) High expression of FUS in HCC tissues (<https://www.proteinatlas.org/ENSG00000089280-FUS/pathology/tissue/liver+cancer#img>). (y) RACGAP1 protein was not detected in normal liver tissues (https://www.proteinatlas.org/ENSG00000161800-RACGAP1/tissue/liver#img). (z) Moderate expression of RACGAP1 in HCC tissues (https://www.proteinatlas.org/ENSG00000161800-RACGAP1/pathology/tissue/liver+cancer#img).
